# Measurement of complement proteins may aid interpretation of pathophysiology in kidney transplant recipients with atypical hemolytic uremic syndrome

**DOI:** 10.1007/s13730-026-01114-5

**Published:** 2026-04-18

**Authors:** Masayoshi Miura, Hiroshi Higashiyama, Takahiro Tsuji, Katsuki Ohtani, Nobutaka Wakamiya

**Affiliations:** 1 Department of Urology and Renal Transplantation, Sapporo Hokuyu Hospital, 5-1, Higashi-Sapporo 6-jo 6-chome, Shiroishi-ku, Sapporo, 003-0006 Japan; 2 Department of Pathology, Sapporo City General Hospital, Sapporo, Japan; 3 Department of Clinical Nutrition, Rakuno Gakuen University, Ebetsu, Japan; 4 The Japanese Association for Complement Research, Osaka, Japan; 5 Department of Medicine and Physiology, Rakuno Gakuen University, Ebetsu, Japan

**Keywords:** Atypical hemolytic uremic syndrome, Kidney transplantation, Complement, Eculizumab

## Abstract

A 53-year-old woman with kidney failure and low-titer donor-specific antibody underwent a living-unrelated donor kidney transplant. Presentation of severe thrombotic microangiopathy (TMA) resulted in early graft loss at day 8 post-transplant and initiation of hemodialysis. Thorough examination of the medical data revealed the patient to have had atypical hemolytic uremic syndrome (aHUS). Hemolytic anemia persisted for several months, even after complete embolization of the kidney graft. The patient subsequently received prophylactic eculizumab treatment and underwent a successful second transplant from a familial donor. Withdrawal of eculizumab resulted in development of TMA in the second graft, while reintroduction of eculizumab reversed the TMA. We examined complement-related profiles over time, and serial measurement of factors such as factor Ba, soluble C5b-9, and C3, and of total complement activity (CH50), clearly reflected the underlying pathophysiological condition and treatment status of the patient before, during, and after transplant. Longitudinal observation of complement protein levels may therefore aid in identifying candidates for kidney transplant who are at risk of aHUS, and monitoring patient status during transplant and follow-up.

## Introduction

Atypical hemolytic uremic syndrome (aHUS) is a condition with multifactorial causes, including abnormalities in the regulation of the complement system [1]. In many patients, genetic abnormalities in complement components have been identified [2, 3], but up to 40% of patients have no detectable causative mutations [4]. Recurrent cases of unidentified aHUS after kidney transplantation have been reported [5] and the use of eculizumab may prevent catastrophic results [6, 7]. Herein we report a case of a kidney transplant recipient who lost function of her first kidney graft after rapid onset of aHUS, but who successfully underwent a second transplant after prophylactic treatment with eculizumab. Furthermore, clear changes in the activity of the complement system throughout the clinical course were evident, which were found to have a marked correspondence with the ongoing pathology, histopathologic data, and treatment regimen administered.

## Case report

A 53-year-old woman with kidney failure was referred to our center to undergo a living-unrelated kidney transplantation. The kidney disease was attributed to historical preeclampsia, with an onset 20 years prior, although exact details were unclear. The patient’s serum creatinine (sCr) levels were approximately 4.0 mg/dL, corresponding to an estimated glomerular filtration rate of 10 mL/min/1.73 m^2^. In addition, warfarin was being administered to the patient because of a history of mitral valve replacement for infectious endocarditis.

The first donor was an unrelated living male. We recorded three human leukocyte antigen (HLA) mismatches and a compatible ABO blood type between the donor and recipient. Although the patient was found to have donor-specific antibodies (DSA) against HLA-B51 with a mean fluorescence intensity of 1500 (Labscreen, One Lambda, USA), the T-cell and B-cell flow cytometry crossmatches were negative.

**Fig. 1:**
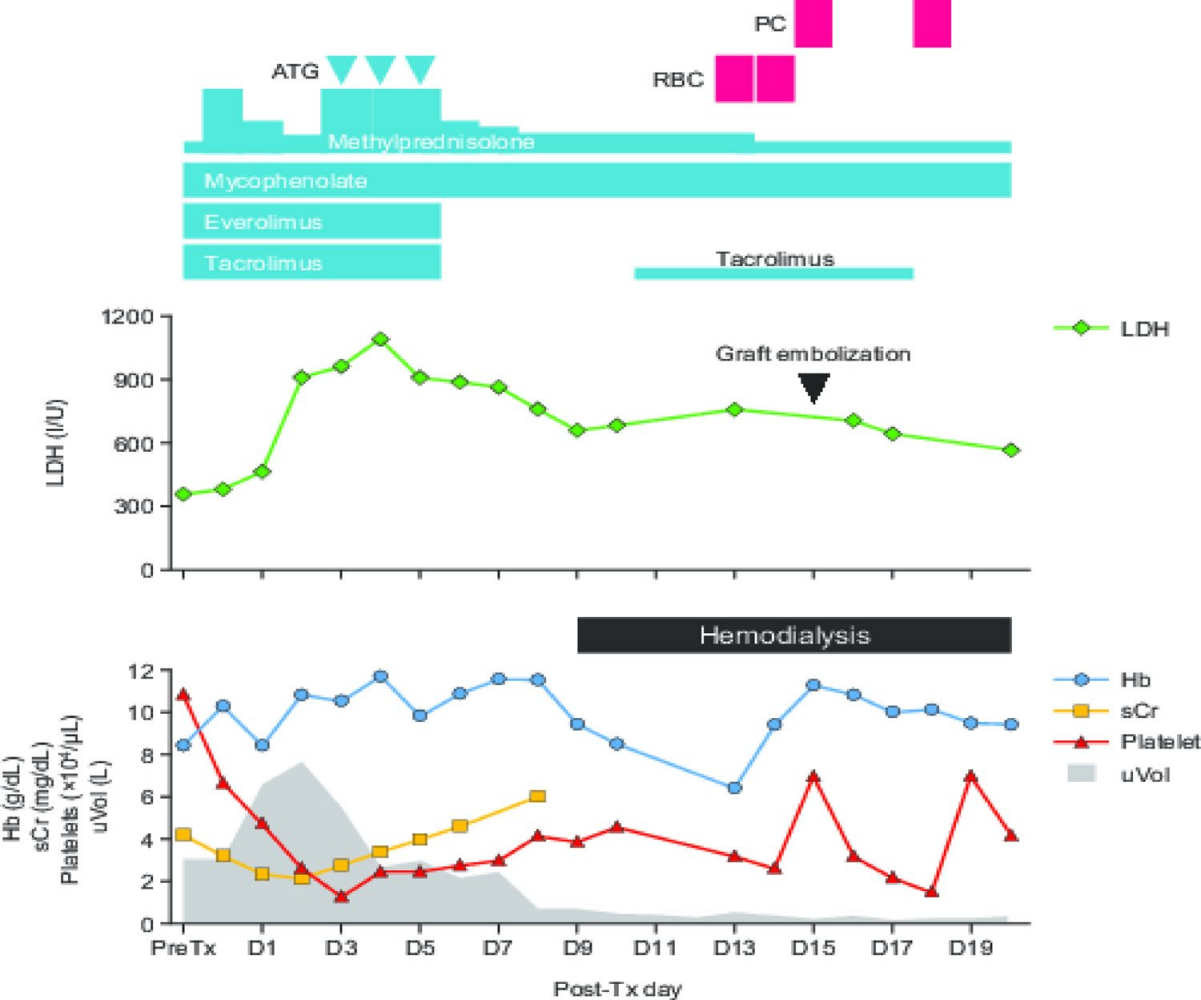
Clinical course after the first kidney transplant. The treatment regimen and laboratory test data are shown over time post-transplant. ATG, anti-thymocyte globulin; D, day; Hb, hemoglobin; LDH, lactate dehydrogenase; PC, platelet concentrate transfusion; RBC, red blood cell transfusion; sCr, serum creatinine; Tx, transplant; uVol, urine volume

**Fig. 2:**
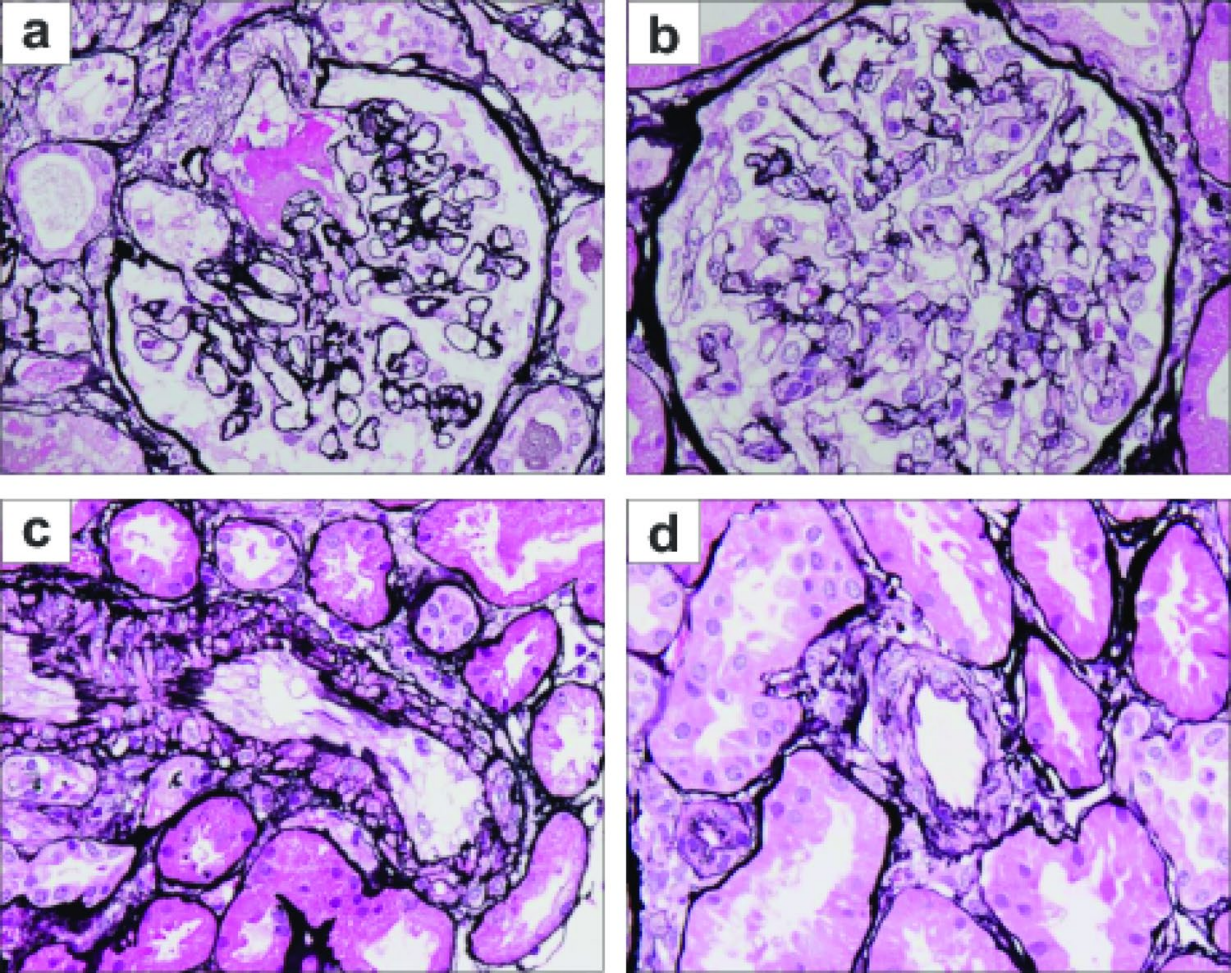
Kidney allograft biopsy findings (**a**) Biopsy findings 9 days after the first kidney transplant; thrombus formation in the glomerular hilar blood vessels showed evidence of TMA, (**b**, **c**) Biopsy findings 9 days after the second transplant; in the glomerulus, swelling of endothelial cells and endocapillary hypercellularity were found (**b**) and significant endothelial cell enlargement was observed in the interlobular arteries, suggesting TMA (**c**). (**d**) Biopsy findings 1 month after the second transplantation; endothelial cell enlargement of the interlobular arteries has disappeared. All panels show periodic acid-methenamine-silver stain specimens. Images were taken with a 40× objective lens. TMA, thrombotic microangiopathy

We started our standard desensitization regimen for DSA-positive, flow cytometry crossmatch-negative cases, comprising tacrolimus (TAC), mycophenolate mofetil, everolimus (EVR), and corticosteroids, 4 weeks before the planned transplant. Warfarin was replaced with intravenous heparin 1 week before transplant. Two doses of basiliximab were given as an induction antibody treatment. During the transplant surgery, immediate urine output was observed after reperfusion of the grafted kidney. However, the graft became soft within 30 min. After excluding all possible surgical problems, we took a biopsy of the graft 2 h after reperfusion and continued close observation of the post-transplant clinical course. This was complicated with severe thrombocytopenia on day 1, with further deterioration, although sCr levels decreased and urine output was maintained (Fig. 1). Histopathologic examination of the biopsy sample showed the presence of thrombotic microangiopathy (TMA). We initially suspected acute antibody-mediated rejection as the cause of the TMA, possibly because of DSA adsorption within the graft; however, DSA was undetectable at day 2. We administered three doses of bolus methylprednisolone, and anti-thymocyte globulin as an anti-rejection regimen. Despite this, elevations in sCr were observed after day 3, and urine output deteriorated. Ultrasonic examination showed progressive deterioration of graft perfusion, while laboratory tests indicated that lactate dehydrogenase levels had increased. At day 8, the graft perfusion had become almost undetectable, and at day 9, the graft biopsy showed severe TMA and diffuse cortical necrosis (Fig. 2a). At this time, attempts to rescue the graft were stopped, and hemodialysis was started. In addition to thrombocytopenia, the patient showed evidence of progressive anemia, and signs of hemolysis (including decreased haptoglobin and fragmented erythrocytes), which were considered compatible with the presence of hemolytic uremic syndrome (HUS). Because we assumed the focus of TMA had been in the allograft, we performed ethanol embolization of the kidney graft. However, the HUS status persisted even after complete graft embolization, indicating that the TMA was systemic rather than localized to the graft. After restarting warfarin, platelet counts slowly recovered, but hemolytic anemia persisted for several months (until the second transplant), requiring periodic blood transfusion despite administration of erythropoiesis-stimulating agents at the full recommended dose.

After a full review of the patient’s medical data, a final diagnosis of aHUS was determined. Laboratory testing indicated a negative result for Shiga toxin, normal ADAMTS13 activity, and a negative result for ADAMTS13 inhibitor. Other causes of secondary TMA, such as post-transplant antibody-mediated rejection, were excluded. Drug-related TMA was considered, but the trough levels of everolimus (below 2 ng/mL when measured before transplantation) and tacrolimus (5.2 ng/mL before transplantation, 2.5 ng/mL on day 13, and 2.5 ng/mL on day 18) precluded this. The persistence of TMA after discontinuation of TAC and EVR also excluded the possibility of drug-related TMA. Immunohistochemical staining for complement components C1q, C3, C4, and C4d was performed. For C1q, C3, and C4, the results were negative; for C4d, the expression was determined to be minimal and not clinically significant. Analyses of complement-related genes failed to detect any known abnormality causative of aHUS, and the patient was also negative for anti-complement factor H antibody. Thus, although no pathogenic variants in complement-related genes or anti-factor H antibodies were identified, all other causes of TMA were excluded, and the condition was therefore diagnosed as clinical aHUS.

Subsequently, a second transplant was planned, with a related living female as the second donor. The second donor was haplotype mismatched and ABO incompatible (B to O). The anti-B antibody level was very low, with both IgG and IgM titers of 1:2. No DSA was detected.

**Fig. 3:**
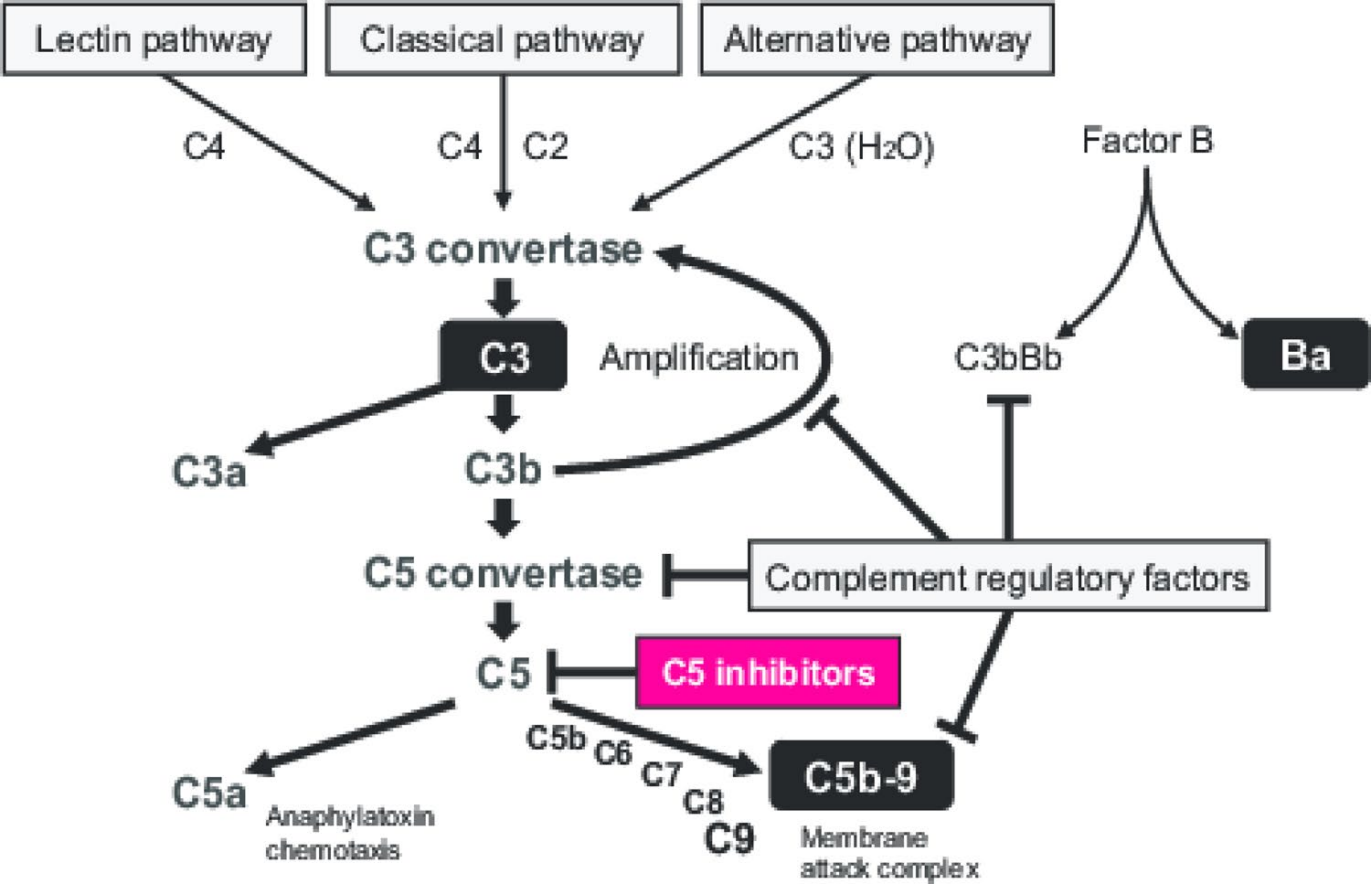
Schematic of the complement system. Components of the complement system are shown schematically. C3 plays a central role in the amplification of the complement system; it is cleaved by C3 convertase, resulting in production of C3b, which can covalently bind to cell surface carbohydrates or immune aggregates. In turn, C3b activates C5 convertase, resulting in production of the terminal complement complex, sC5b-9 (also known as the membrane attack complex). Factor Ba is the fragment of complement factor B that results from activation of the alternative pathway. The factors measured in this study are shown in black boxes with white lettering

**Fig. 4:**
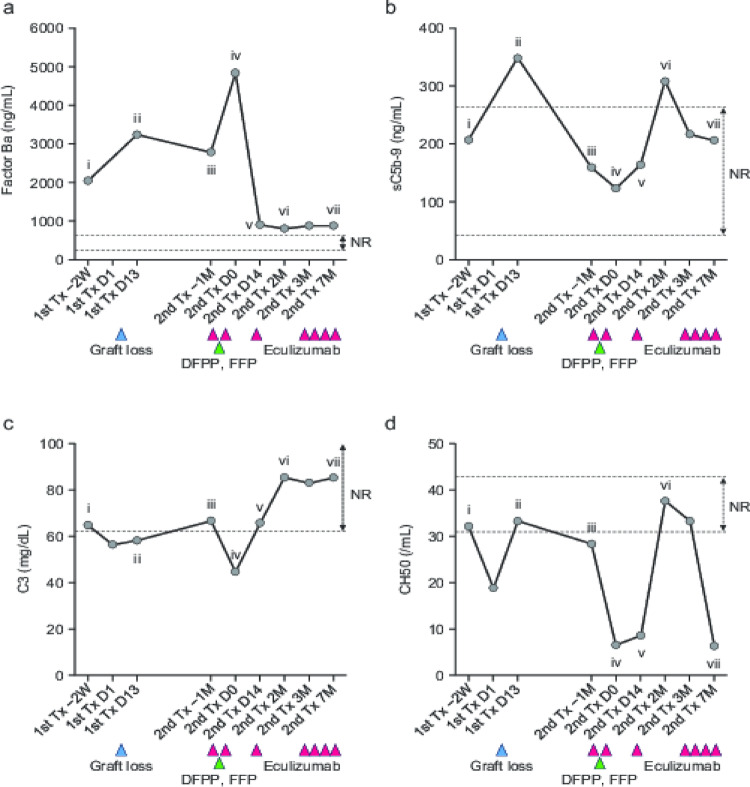
Changes in complement components and complement-related factors. Changes in the levels of factor Ba (**a**), sC5b-9 (**b**) and C3 (**c**), and CH50 (**d**) are shown, along with clinical condition and treatment effects; specific timepoints are labeled with Roman numerals. Blue triangles indicate graft loss, red triangles represent administration of eculizumab, and green triangles denote treatments with DFPP and FFP. Dotted lines and arrows show normal ranges for each component (mean ± 2 standard deviations): factor Ba, 275.6–685.2 ng/mL; sC5b-9, 37.0–260.6 ng/mL; C3, 61.3–131.7 mg/dL; CH50, 31.2–43.2 CH50/mL. D, day; DFPP, double filtration plasmapheresis; FFP, fresh frozen plasma; M, month; NR, normal range; Tx, transplant; W, week

The desensitization regimen was identical to that of the first transplant, with the addition of plasmapheresis to remove any possible abnormal complement-related proteins or to supplement any defect. Furthermore, eculizumab (a C5 inhibitor; Fig. 3) was administered 2 weeks before the transplant, and again after plasmapheresis on the day before transplant (Fig. 4). The clinical course immediately after the second transplant was uneventful, without any clinical signs of TMA. However, a slight elevation in sCr necessitated a biopsy of the second graft on day 9, and histopathologic analysis indicated the presence of weak TMA (Fig. 2b and c). Development of weak TMA under terminal complement inhibition may have been mediated by antibody-dependent cytotoxicity, given the recent history of aHUS. Nevertheless, because we could not definitively exclude acute T-cell-mediated rejection, bolus methylprednisolone and antithymocyte globulin were administered. Thereafter, sCr levels gradually improved; however, total complement activity (CH50) increased at day 14, and an additional dose of eculizumab was administered (Fig. 4d). At 1 month post-transplant, allograft biopsy showed normal histology and eculizumab was discontinued (Fig. 2d).

At 4 months post-transplant, the patient presented with elevated sCr levels, accompanied by histological evidence of TMA in a biopsy sample. Eculizumab treatment was restarted and the clinical course remained stable. Biopsies obtained at 1 year and 3 years post-transplant showed no evidence of TMA; at 4 years, the functionality of the grafted organ showed no deterioration, with a sCr level of 1.45 mg/dL.

### Changes in complement-related factors

Using cryopreserved plasma obtained from the patient, we measured the following complement-related factors: factor Ba, soluble C5b-9 (sC5b-9), and C3 (Fig. 3), along with CH50 (a measure of classical complement pathway activity). Two weeks prior to the first transplant, the factor Ba level was elevated, indicating the presence of natural activation of the alternative pathway (Fig. 4a, i); at the same time point, the sC5b-9 level was within the normal range and the terminal cascade was regulated (Fig. 4b, i). At day 13 after the first transplant, when TMA was very active, both factor Ba and sC5b-9 levels were elevated, indicating activation of both the alternative and terminal complement pathways (Fig. 4a, ii and 4b, ii). A slight reduction in the C3 level indicated amplification of the alternative pathway, resulting in consumption of C3 (Fig. 4c, ii). Hemodialysis was continued for 10 months after the loss of the first graft, during which time the factor Ba level remained high (Fig. 4a, iii) and hemolytic anemia was persistent (data not shown).

Eculizumab (900 mg) was administered 14 days and 1 day prior to the second transplant. On the day of the second transplant, the factor Ba level remained high (Fig. 4a, iv), but sC5b-9 and CH50 levels were reduced (Fig. 4b, iv and Fig. 4d, iv). At day 14 after the second transplant, a further dose of eculizumab (600 mg) was administered. At this time, the patient was in a stable condition, and sC5b-9 was within the normal range (Fig. 4b, v). At 2 months after the second transplant, both sC5b-9 and CH50 levels were elevated (Fig. 4b, vi and Fig. 4d, vi). At 4 months after the second transplant, histopathologic evidence of TMA was observed in the graft (data not shown), and eculizumab treatment was reinitiated. At 7 months after the second transplant, the C5b-9 level had normalized and the CH50 level was suppressed (Fig. 4b, vii and Fig. 4d, vii); corresponding histopathologic data indicated that signs of TMA in the graft had improved (Fig. 2d).

## Discussion

In summary, we have presented a case in which a patient with kidney failure lost a first graft due to aHUS, but underwent a second successful kidney transplant with a treatment regimen involving the use of eculizumab prophylaxis and maintenance therapy. In addition, we have shown a clear link between complement activation and the clinical course during and after the first and second transplant procedures.

Although aHUS is a rare disorder, it is linked to graft failure and poor clinical outcomes following kidney transplantation [6]. In this case, the first graft became soft within 30 min of reperfusion, TMA was visible on histopathologic sections at 2 h post-transplant, and deterioration continued despite rescue therapy, with hemodialysis initiated on day 9. For the second transplant, it was decided to add prophylactic eculizumab to the pre-transplant desensitization regimen. Since eculizumab was first approved for the treatment of aHUS in the US in 2011 [8], substantial evidence has accumulated supporting its use as prophylactic and/or maintenance therapy in patients undergoing kidney transplantation [6]. Eculizumab is an inhibitor of complement C5 cleavage, which prevents the assembly of C5b-9 and subsequent endothelial injury [9]; prophylactic treatment improves renal allograft survival [6, 10, 11], while maintenance treatment improves hematologic parameters and renal function [9, 11]. In our patient, prophylactic eculizumab allowed successful transplantation, with no signs of TMA for the first 9 days. Subsequent development of weak TMA required the initiation of ongoing maintenance eculizumab, and the patient has subsequently remained stable with no evidence of TMA and good organ function. Another center has reported good long-term outcomes after a decade of follow-up, with a high rate of graft survival and no serious infections [12].

In addition, we found that there were obvious discernible changes in complement components and complement-related factors over the clinical course that clearly reflected the pathophysiological condition and treatment effects. An abnormal circulating complement profile has been reported in up to two-thirds of patients with aHUS [13], and the observed elevations in levels of Ba and sC5b-9 at the time of graft loss after the first transplantation suggest their potential utility as markers reflecting the disease status at the time of kidney transplantation. In addition, C5b-9 endothelial deposition has been suggested as a marker for monitoring eculizumab efficacy [13, 14]. In this case, administration of eculizumab prior to the second transplantation led to a further decrease of sC5b-9, which had been elevated, into the normal range, and strong suppression of CH50. We consider that both findings reflect the inhibition of terminal complement activity by eculizumab. In contrast, from day 14 after the second transplant (eculizumab administration) to 2 months after the second transplant (i.e., approximately 1.5 months later), sC5b-9 rose above the upper limit of the normal range, and CH50 increased to within the normal range. These results may reflect a weakening of C5 inhibition due to a decline in the serum concentration of eculizumab. Based on these temporal changes, sC5b-9 and CH50 may serve as useful, and sensitive, markers for monitoring the therapeutic effect of eculizumab. Overall, our data suggest that observation of complement-related protein levels may aid understanding of the pathological condition before, during, and after transplant, as well as provide a method to detect kidney transplant candidates at potential risk of aHUS.

In conclusion, although our data were from a single patient, the clinical outcome with eculizumab aligns with published data. The corresponding analysis of complement factors strongly suggests an area of research that warrants further investigation.
